# Impact of Land Use and Land Cover (LULC) Changes on Carbon Stocks and Economic Implications in Calabria Using Google Earth Engine (GEE)

**DOI:** 10.3390/s24175836

**Published:** 2024-09-08

**Authors:** Yasir Hassan Khachoo, Matteo Cutugno, Umberto Robustelli, Giovanni Pugliano

**Affiliations:** 1 Department of Engineering, University of Naples Parthenope, 80143 Naples, Italy; yasirhassan.khachoo001@studenti.uniparthenope.it (Y.H.K.); umberto.robustelli@uniparthenope.it (U.R.); 2 University of Benevento Giustino Fortunato, 82100 Benevento, Italy; 3 Department of Civil, Architectural and Environmental Engineering, University of Naples Federico II, 80125 Naples, Italy; giovanni.pugliano@unina.it

**Keywords:** LULC, carbon sequestration, CO_2_ emissions, Calabria, remote sensing, GEE, InVEST

## Abstract

Terrestrial ecosystems play a crucial role in global carbon cycling by sequestering carbon from the atmosphere and storing it primarily in living biomass and soil. Monitoring terrestrial carbon stocks is essential for understanding the impacts of changes in land use on carbon sequestration. This study investigates the potential of remote sensing techniques and the Google Earth Engine to map and monitor changes in the forests of Calabria (Italy) over the past two decades. Using satellite-sourced Corine land cover datasets and the InVEST model, changes in Land Use Land Cover (LULC), and carbon concentrations are analyzed, providing insights into the carbon dynamics of the region. Furthermore, cellular automata and Markov chain techniques are used to simulate the future spatial and temporal dynamics of LULC. The results reveal notable fluctuations in LULC; specifically, settlement and bare land have expanded at the expense of forested and grassland areas. These land use and land cover changes significantly declined the overall carbon stocks in Calabria between 2000 and 2024, resulting in notable economic impacts. The region experienced periods of both decline and growth in carbon concentration, with overall losses resulting in economic impacts up to EUR 357.57 million and carbon losses equivalent to 6,558,069.68 Mg of CO_2_ emissions during periods of decline. Conversely, during periods of carbon gain, the economic benefit reached EUR 41.26 million, with sequestered carbon equivalent to 756,919.47 Mg of CO_2_ emissions. This research aims to highlight the critical role of satellite data in enhancing our understanding and development of comprehensive strategies for managing carbon stocks in terrestrial ecosystems.

## 1. Background and Related Works

### 1.1. Background

Ecosystem Services (ESs) refer to the benefits and effectiveness of ecological processes for human well-being [1]. Changes in Land Use and Land Cover (LULC) greatly influence the supply and value of various ESs, particularly climate regulation services, due to fundamental alterations in the structure and function of forests, agriculture, and other ecosystems over time [2]. These spatio-temporal LULC changes can lead to an increase in the provision and value of some services while simultaneously decreasing others [3], especially in arid regions. These regions, which are highly vulnerable to human disturbance, encompass about 40% of the Earth’s land surface [4]. Although often perceived as unproductive, these ecosystems provide essential ESs crucial for sustaining well-being, such as Carbon Sequestration (CS) [5]. CS involves the long-term storage of carbon in soil, plants, and other forms to mitigate or slow down climate change [6]. In this context, terrestrial ecosystems (such as forests, orchards, and agricultural systems) and aquatic ecosystems (like water bodies and wetlands) play a critical role in carbon cycling [7]. Among terrestrial ecosystems, agriculture often acts as a source of greenhouse gases [8], whereas forests and orchards typically sequester atmospheric carbon [9]. Due to their structure, long life cycle, and minimal soil disturbance, orchards can store substantial amounts of atmospheric carbon [10]. Generally, the forest carbon stock refers to the amount of carbon sequestered from the atmosphere through photosynthesis and currently retained within the forest ecosystem, primarily within living biomass and soil, with smaller amounts found in dead wood and litter. Forests are pivotal in the global carbon cycle, influencing the climate on a worldwide scale [11,12]. Countries, in accordance with the United Nations Framework Convention on Climate Change (UNFCCC), rely on national forest inventory data to gauge carbon absorption linked to changes in land use and forestry [13]. The UNFCCC and Kyoto Protocol prioritize five primary terrestrial carbon reservoirs: soil, litter, Above-ground Biomass ($B_{AG}$), Below-ground Biomass ($B_{BG}$), and deadwood. Conversely, the degradation of forests and orchards can exacerbate climate change [14]. The impact of escalating climate change is directly linked to the supply and value of climate regulation services, and it is estimated that approximately 50% of the total monetary value of major terrestrial biomes (including forests, grasslands, and woodlands) is attributed to climate regulation services [15]. The rapid decline in forest cover and the increase in forest degradation are primarily due to land clearing, burning, and overexploitation. According to a recent Food and Agriculture Organisation’s report, global forest areas decreased by 178 million Hectares (ha) from 1990 to 2020, causing approximately 12–20% of total global emissions, making it the second-largest source of emissions worldwide [16]. In response to these challenges, several international agreements have been established. One notable agreement is the Reducing Emissions from Deforestation and forest Degradation (REDD+) initiative under the UNFCCC [17]. REDD+ is a results-based financial scheme aimed at reducing carbon emissions or enhancing carbon removals in exchange for financial compensation. This compensation is contingent upon the monitoring, measurement, and verification of REDD+ activities, ensuring emissions are below the Forest Reference Emission Level (FREL). To support these efforts, robust methods for Monitoring, Reporting, and Verifying (MRV) carbon emissions, reductions, or removals are essential. Hence, forest inventories are commonly used for MRV purposes, including the establishment of FREL, which serves as a benchmark for assessing emission reductions or removals for financial incentives [18].

### 1.2. Relevant Works

It is worth noting that, until a few decades ago, forest monitoring primarily depended on on-site surveys, which were not only costly but also time- and energy-consuming, even when high accuracy was not a significant concern [19]. Past field surveys were confined to assessing tree heights, tree counts, and canopy coverage percentages using fixed plots [20,21]. A significant advancement in forest monitoring was the introduction of aerial photography [22]. However, the poor accuracy of optical sensors used in the past hindered substantial progress. Nowadays, advancements in Remote Sensing (RS) technology, particularly through high-resolution satellite imagery, enable the tracking of gradual or sudden alterations in forest cover [23]. The availability of such images in convenient time-series formats has elevated RS to the foremost method for efficient forest monitoring and change detection [24,25]. To estimate forest carbon, various satellite missions provide essential optical imagery. Multispectral imagery from Landsat missions, high-spectral-resolution hyperspectral imagery such as EO-1 Hyperion, high-resolution imagery such as WorldView-3, and 3D structural data with optical imagery such as LiDAR-integrated imagery from Sentinel-2 could enhance biomass and carbon stock estimates by allowing the precise identification and detailed mapping of tree species, as well as disturbance detection. Nevertheless, for RS-based research to have global applicability, satellite images from diverse sensors must be readily accessible, free of charge, and easily obtainable for each region at acceptable spatial and temporal resolutions. This poses a challenging and costly task that requires massive computational and storage capacity. In response to this need, various cloud-based platforms equipped with high-performance computing capabilities to facilitate online RS dataset processing were developed over time and include Amazon Web Services, which contains a comprehensive suite of tools for processing large satellite datasets with scalable options and direct satellite data access; Google Earth Engine (GEE), which simplifies geospatial analysis with its vast data catalog and cloud-based processing and is ideal for rapid assessments; Sentinel Hub, which specializes in the efficient access and processing of Sentinel data and is perfect for high-resolution monitoring; Microsoft Azure’s Planetary Computer, which integrates AI with geospatial data for large-scale environmental research; IBM Cloud with Watson, which provides advanced AI tools for geospatial analysis; and DeepESDL, which focuses on multi-dimensional data and deep learning for integrated environmental research. The choice depends on the data needs, the scale of the analysis, and computational requirements. This study utilizes GEE for its vast data catalog and cloud-based processing, launched on 2 December 2010 [26]. The GEE platform allows users to perform geospatial analysis on Google’s infrastructure through various methods, including the Code Editor, a web-based integrated development environment for scripting, and the Explorer, a lightweight web app for data exploration and simple analyses. Additionally, GEE offers client libraries with JavaScript and Python wrappers around the Earth Engine application programming interface, enabling the creation of custom applications and the local development of Earth Engine code, with numerous demos available in the GitHub repository. To address the challenges of monitoring and mitigating climate change, it is crucial to understand the carbon dynamics within our planet’s ecosystems. While the terrestrial biosphere, ocean, and atmosphere serve as carbon reservoirs, with Carbon dioxide (CO_2_) naturally cycling through these systems, they are currently unable to absorb all anthropogenic CO_2_ emissions. According to Prentice et al. [27], after the industrial era, atmospheric concentrations of CO_2_ have risen continuously primarily due to anthropogenic emissions from fossil fuel burning, which accounted for about three-quarters of the emissions. This escalating atmospheric CO_2_ concentration causes global warming and degrades ESs and biodiversity, thereby impacting both human life and ecological health [28]. To mitigate this accumulation, it is essential to enhance natural carbon pools and create new carbon sinks through terrestrial CS, which involves transferring atmospheric CO_2_ into biotic and pedologic carbon pools; this process occurs when the natural uptake of carbon by plants and soils surpasses the losses from plant and soil respiration and biomass removal, consequently storing CO_2_ in vegetation and soils, both above- and below-ground, known as phyto-sequestration and soil CS, respectively [29]. Various international scientific groups have recommended the Integrated Valuation of Ecosystem Services and Tradeoffs (InVEST) model as an advanced tool for assessing ES and quantified and mapped carbon storage and sequestration [30,31,32]. Studies have shown that as an important component of global carbon storage and emissions, terrestrial ecosystems play an important role in mitigating climate change [33,34]. The CS capacity of different LULC types exhibits significant differences. For instance, Zhang et al. [35] showed that the change in LULC caused a loss of 279 Teragram (Tg) of carbon storage in China’s terrestrial ecosystems from 1980 to 2010. Therefore, it is efficient to understand the characteristics of regional carbon storage and carbon balance changes from the perspective of LULC types, including the changes in carbon balance caused by LULC structural changes. Additionally, it is important to consider carbon storage potential under various future scenarios and the impact of land resource management on carbon balance. Furthermore, in most LULC studies, the accuracy assessment and validation of LULC classifications and predictive models are performed using kappa parameters, which serve as a measure of agreement between the simulated and reference maps. According to Sim and Wright [36], a kappa parameter ranging from 0.0 to 0.20 indicates slight agreement, 0.21 to 0.40 suggests fair agreement, 0.41 to 0.60 reflects moderate agreement, 0.61 to 0.80 indicates substantial agreement, and a value greater than 0.80 signifies almost perfect agreement.

### 1.3. Overview of the Study

This study investigates the potential of RS techniques and GEE for mapping and monitoring purposes at a regional scale. To validate this procedure, the region of Calabria has been selected due to the diversity of its landscapes. It focuses on mapping and monitoring changes in the LULC of Calabria and their associated CS capacities over the past 18 years. Firstly, it quantifies the amount of carbon currently stored in the Area of Interest (AOI) and explores the correlation between changes in LULC and CS capacity. It assesses the extent of gain or loss in fixed carbon, using species specific to the Calabrian environment. Additionally, it develops a valuation model for carbon pools at different times, utilizing LULC information, the monetary value of carbon, and an annual discount rate of carbon. The innovative aspect of this study lies in its combination of remotely sensed datasets, the InVEST model, and GEE to explore the capacity of Calabrian landscapes to sequester and store carbon over time.

## 2. Materials and Methods

### 2.1. Study Area

**Figure 1:**
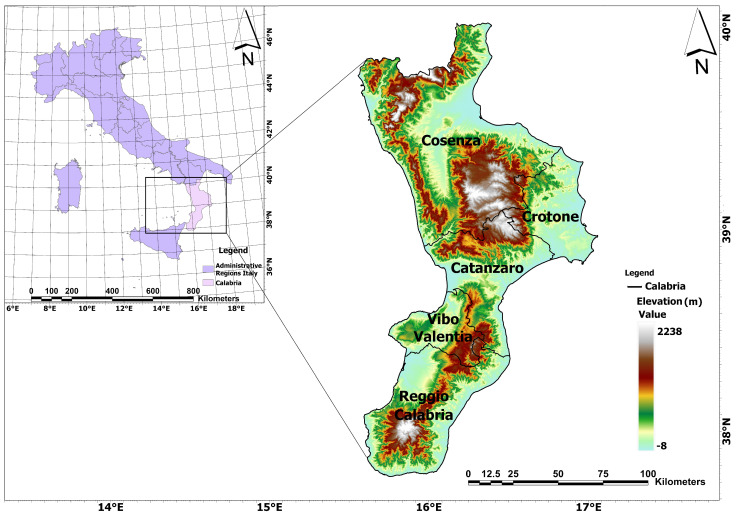
Location of the AOI: The Italian peninsula with regional boundaries on the left, and a detailed, zoomed—in view of Calabria on the right, reporting its provinces and Digital Elevation Model (DEM).

The Calabria region lies in the southern part of the Italian peninsula, covering approximately 15,118 km^2^, as reported in Figure 1. Over 90% of its terrain is characterized by mountains and hills, including the massif of Pollino, the plateau of Aspromonte, and the massif of Sila [37]. The region boasts nearly 800 km of coastline along the Ionian Sea and the Tyrrhenian Sea, accompanied by numerous streams [38]. Geologically, Calabria is diverse, featuring granite rocks in the Sila mountains, metamorphic formations in the coast heights, and dolomite limestone in the Pollino massif [39]. The Mediterranean climate prevails, with mild winters and hot summers, accompanied by an annual average rainfall of 1176 mm [40]. Despite the overall rainfall, the region experiences a minimum drought season lasting at least three months. Calabria’s climate is temperate, influenced by factors such as elevation, proximity to the sea, prevailing winds, and orientation. Moreover, Calabria’s diverse regions are characterized by beech, larch pine, and loricate pine in the Sila Plateaus; chestnut in Reventino; olive, citrus, mulberry, fig, and grapevine on the Violet Coast; monumental olive trees in Gioia Tauro; bergamot in the Bergamot Plain; and citron in the Riviera dei Cedri, each reflecting the area’s cultural and agricultural heritage [41]. This study examines the landscapes of Calabria, Italy, offering valuable insights into terrestrial CS and land use alterations within a Mediterranean context. A detailed carbon pool table has been specifically tailored for Calabrian forests, taking into account the three predominant tree species and the average values for various crops cultivated in the area.

### 2.2. Data Acquisition

The LULC data are sourced from the Copernicus Land Monitoring Services, commonly recognized as CORINE Land Cover (CLC) [42], for the years 2000, 2006, 2012, and 2018. The datasets were obtained and processed with a JavaScript code developed in GEE for this purpose. For areal phenomena, CLC adopts a Minimum Mapping Unit of 0.25 km^2^, while for linear phenomena, a minimum width of 100 m is employed. The datasets offer a visualization of general land cover patterns at a scale of 1:100,000 and employ the European Terrestrial Reference System 1989. The national CLC databases are produced by the European Environment Information and Observation Network, National Reference Centres Land Cover, coordinated and integrated by the European Environment Agency. In most countries, CLC is developed through the visual interpretation of high-resolution satellite data [43]. Some nations employ semi-automatic methodologies, utilizing a combination of national in situ data, satellite image processing, Geographical Information System (GIS) integration, and generalization techniques.

### 2.3. Data Processing

**Figure 2:**
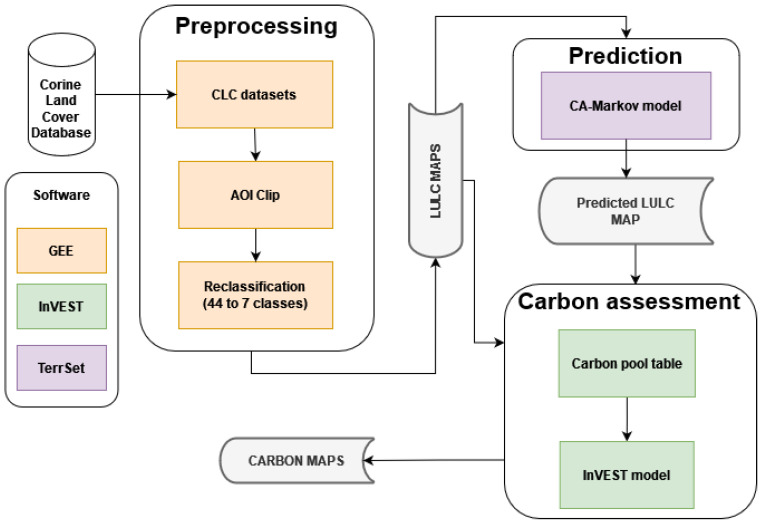
Methodological flowchart: the preprocessing stage (yellow) uses GEE for data clipping and reclassification. The prediction stage (purple) employs the CA–Markov model in TerrSet to generate future land use maps. The carbon assessment stage (green) utilizes InVEST to estimate carbon stocks based on land use maps. Each color corresponds to a different software used in that stage.

As indicated in Figure 2, the methodology comprised three main phases: preprocessing, prediction, and carbon assessment, each of which utilized specific software tools. Preprocessing was performed in GEE (Google, Mountain View, CA, USA), the prediction phase was conducted in TerrSet 19.0.6 (Clark Labs, Worcester, MA, USA), and InVEST 3.14.1 (Natural Capital Project, Stanford University, Stanford, CA, USA) was used for carbon assessment. In the preprocessing phase, the LULC datasets were acquired and clipped to match the AOI. The datasets were then reclassified to broader classes, streamlining the data for more efficient analysis. During the prediction phase, the reclassified LULC maps were used as inputs for the CA–Markov model, which forecasted future LULC scenarios. In the carbon assessment phase, the LULC maps were utilized in combination with a carbon pool table to estimate the carbon storage. The carbon pool table was required to assign carbon values to each LULC class. The details of each step are described in the following subsections.

#### 2.3.1. Preprocessing

The initial phase involved the clipping procedure, which aimed to restrict the AOI to Calabria within the CLC datasets of Europe for the years 2000, 2006, 2012, and 2018. Subsequently, a reclassification was executed using the guidelines provided by the Intergovernmental Panel on Climate Change (IPCC) [44], employing a reclassifying operator in GEE. In addition to the classes defined by Eggleston et al. [44], an additional class of water bodies was included in the classification based on the literature [45] and the distinction between water body and wetland characteristics. In this process, a total of 44 classes in the CLC datasets were transformed into 7 new classes: settlement, cropland, forest land, grassland, wetland, water bodies, and other land. Table 1 provides the correspondence between these 7 reclassified classes and the original 44 CLC classes, along with concise descriptions.

#### 2.3.2. LULC Processing

In the second step, a systematic methodology was employed as described by IPCC 2006 [44,45] to perform LULC calculations for the years 2000, 2006, 2012, and 2018 [46]. The primary objective of the LULC mapping process was to provide comprehensive information regarding the spatial distribution of distinct land use categories. Furthermore, the methodology aimed to identify and quantify LULC changes over 6-year intervals, specifically focusing on the temporal evolution from 2000 to 2018. The simulation and projection of the changes in LULC in Calabria between 2000 and 2018 served as the foundation for predicting the future landscape in 2024.

#### 2.3.3. Prediction

Researchers have developed numerous simulation methods to observe future scenarios of LULC. Different techniques were applied in various studies, including the Cellular Automata–Markov chain (CA–Markov) model [47]; mathematical-based spatiotemporal modeling [48]; system dynamic simulation [49]; statistical, cellular, and hybrid models [50]; and cellular- and agent-based models or a hybrid of the two [51]. Model parametrization, transition probability computations, neighborhood rule determination, remote sensing, and GIS datasets [52] were all used to construct the initial conditions of the CA–Markov model. The CA–Markov model was recognized as one of the most appropriate and widely accepted methods for Land Use Land Cover Change (LULCC) modeling; it projected LULCC probability for the future date ‘t + 1’ by considering ‘t − 1’ to ‘t’. In this study, ‘t − 1’, ‘t’, and ‘t + 1’ referred to the years 2012, 2018, and 2024, respectively. The integration of the CA–Markov model was particularly valuable for modeling land use changes and was capable of simulating and predicting land cover changes [48]. This combination of CA and Markov models provided a robust method for modeling spatiotemporal dynamics as it combines the statistical strength of Markov Chains with the spatial pattern-simulation capability of CA to forecast changes in LULC. It provides realistic, spatially explicit forecasts by taking into account spatial interdependence, generating probabilistic results, and using historical data for transition probabilities. Furthermore, the model’s adaptability to different study scenarios and contexts is made possible by its customization capabilities [48]. The CA component usually adds a spatial dimension to the Markov model, enabling the simulation and prediction of transitions between multiple land use categories [53]. CA consisted of a collection of identical elements, known as cells, each located in a defined, discrete, and regular spatial area [54]. The core principle of CA is that land use change at each location (e.g., cell) could be influenced by its current state and the changes in surrounding cells [55]. This reflected the dependence of transition rules on neighboring cells. Transition rules were expressed using probabilities that considered only the current state [56]. These rules were based on a probability transition matrix, which contained the likelihood of a pixel changing from one LULC category to another or remaining in its original LULC category [57].

The same CA–Markov model was employed in this study to predict the LULC of Calabria for 2024. The predictive model relied on a transition probability matrix that encapsulated the probability that each LULC class would transition to another class. Firstly, three transition probability matrices were calculated by the cross-tabulation of LULC maps for the periods 2000–2006, 2006–2012, and 2012–2018. An average probability matrix was then derived by averaging these three matrices. By considering three transition periods and calculating an average transition probability matrix, the model gains increased in robustness and accuracy in predicting future LULC changes. This approach captures a broader range of land use dynamics over multiple time intervals, reducing the influence of anomalies or short-term fluctuations specific to any single period. As a result, the model provides a more reliable and representative projection of future land use changes, leading to better-informed decisions and more accurate assessments of carbon storage potential and other landscape dynamics. This average transition probability matrix, along with the 2018 LULC map, served as input data to the CA–Markov model, which subsequently predicted the LULC for 2024. The CA–Markov model used a conventional 5 × 5 contiguity filter and considered six iterations of CA, one iteration for each future year.

This model’s performance was validated through two tests using the validation tool in TerrSet 19.0.6:

- First validation test: The LULC datasets for 2000 and 2006 were used with the average probability matrix to predict the LULC for 2012. The predicted LULC for 2012 was then compared with the actual LULC dataset for 2012.
- Second validation test: Similarly, the LULC datasets for 2006 and 2012 were used to predict the LULC for 2018. The predicted LULC for 2018 was validated by comparing it with the actual LULC dataset for 2018.

The validation process generated various kappa parameters, which served as a measure of agreement between the simulated and reference maps. According to Sim and Wright [36], a kappa parameter ranging from 0.0 to 0.20 indicated slight agreement, 0.21 to 0.40 suggested fair agreement, 0.41 to 0.60 reflected moderate agreement, 0.61 to 0.80 indicated substantial agreement, and a value greater than 0.80 signified almost perfect agreement. The average transition probability matrix used in this study is presented in Table 2.

#### 2.3.4. Carbon Storage Assessment

To assess the carbon storage potential of Calabrian landscapes, the InVEST model [58] was used. The InVEST model stood out as a reliable and widely used technique for the quantification of regional carbon storage associated with different LULC types. This model calculated the concentration of carbon within each grid cell of a given region, utilizing a comprehensive carbon density pool. The carbon pool table was constructed based on a rigorous review of Italian greenhouse gas inventory reports, IPCC 2003, 2006, and 2014 guidelines, and other relevant literature [44,45,59,60,61,62,63]. This table delineated carbon distribution as follows: Carbon Above -ground ($C_{AG}$) in $B_{AG}$, Carbon Below-ground ($C_{BG}$) in $B_{BG}$, soil carbon ($C_{SOIL}$), and dead organic matter carbon ($C_{DOM}$) across various LULC classes. For instance, the carbon storage ($C_{l,j,k}$) of a grid cell (j,k) with land use type “l” was given by Aalde et al. [64] as

(1) $$C_{l,j,k}=A\left(C_{{\mathrm{AG}}_{l,j,k}}+C_{{\mathrm{BG}}_{l,j,k}}+C_{{\mathrm{SOIL}}_{l,j,k}}+C_{{\mathrm{DOM}}_{l,j,k}}\right)$$

where *A* is the area of each grid cell in ha. Lastly, the carbon storage $C_{S}$ is estimated using Equation (2) and carbon sequestered *S* can be calculated, using Equation (3) between two years, as follows:

(2) $$C_{S}=\sum_{l=1}^{N}C_{l,j,k}$$

(3) $$S=C^{T2}-C^{T1}$$

where, $C^{T1}$ and $C^{T2}$ correspond to the carbon storage at times T1 and T2 (T2 > T1).

##### Carbon Pools in Forests

By employing growing stock ($G_{S}$) as the primary driver, the forest carbon pools were estimated based on the classifications and definitions set forth in the Good Practice Guidance for Land Use, Land Use Change, and Forestry (LULUCF) [44,59,60]. These carbon pools included $B_{AG}$ and $B_{BG}$ (living biomass), dead wood and litter (dead organic matter), and soil organic matter. The volume of the $G_{S}$ of forests per hectare was obtained from the Italian national forestry inventory reports, which stood at 225.4 $m^{3}/\mathrm{ha}$, available at (http://crea.g3wsuite.it/en/map/consistenza-e-accrescimento/qdjango/27, accessed on 1 March 2024). The $B_{AG}$ per hectare was then calculated using the following equation:

(4) $$B_{AG}=G_{S}\cdot B_{EF}\cdot W_{BD}$$

where $B_{AG}$ is the above-ground biomass in Megagram (Mg) per ha, $G_{S}$ is the volume of growing stock of forests in $m^{3}$ per ha, $B_{EF}$ is the biomass expansion factor, and $W_{BD}$ is the wood basic density in $\mathrm{Mg}\;\mathrm{of}\;\mathrm{dry}\;\mathrm{matter}\;\left(d.m.\right)\;\mathrm{per}\;m^{3}$. The $B_{AG}$ is then converted to carbon content by multiplying with a carbon fraction factor as recommended by IPCC 2006:

(5) $$C_{AG}=B_{AG}\times C_{F}$$

where $C_{AG}$ is the above-ground carbon content in Mg Carbon per ha (MgC/ha), and $C_{F}$ is the carbon fraction factor in MgCperMgd.m. The below-ground carbon content was calculated using the following equation

(6) $$C_{BG}=G_{S}\cdot W_{BD}\cdot C_{F}$$

where, $C_{BG}$ is is the below-ground carbon content in MgC/ha, and *R* is the root-to-shoot ratio, which aids in the conversion of $B_{AG}$ to $B_{BG}$.

$C_{DOM}$ was obtained from the $C_{AG}$ by applying an average mortality rate ($A_{MR}$) of the forest topology as recommended in IPCC 2003. The $A_{MR}$ acted as a dead mass conversion factor and converted the $C_{AG}$ into $C_{DOM}$, as shown below:

(7) $$C_{DOM}=C_{AG}\cdot A_{MR}$$

In addition, the carbon content of litter was calculated from the $C_{AG}$ using regression techniques reported by Federici et al. [65]; therefore, the total $C_{DOM}$ was the sum of the carbon content of deadwood organic matter in the soil and carbon due to litter. Soil organic carbon values in the Calabrian forests were taken from the Italian greenhouse inventory report of 2023. It was assumed in the analysis that the Calabrian forests consisted of four main types of trees: European beech, chestnut, turkey oak, and larches. Hence, a mean value for $B_{EF}$, a mean value for $W_{BD}$, and a mean value for R were considered. Table 3 reports the $B_{EF}$, $W_{BD}$, R, and $A_{MR}$ values, as recommended by IPCC, over time.

##### Carbon Pools Related to Crops

The change in biomass was only estimated for perennial woody crops. For annual crops, an increase in biomass stocks in a single year was assumed to be equal to biomass losses from harvest and mortality in that same year; thus, there was no net accumulation of biomass carbon stocks. Table 4 corresponded to the $C_{AG}$ and $C_{BG}$ found in the woody crops, as reported by the Italian greenhouse inventory report 2023 [63]. An average value for $C_{AG}$ and $C_{BG}$ was then considered for the carbon pools table.

##### Carbon Pools Related to Grassland

Carbon pools in grassland were calculated considering the IPCC 2006 guidelines. The default value of $B_{AG}$ = 2.7 Mg d.m. per ha was obtained from IPCC 2006. The $B_{AG}$ was then converted to $B_{BG}$ by multiplying it by the root-to-shoot ratio, which was equal to 4.0, as reported by IPCC 2006. The $C_{AG}$ (MgC/ha) and $C_{BG}$ (MgC/ha) were then obtained by multiplying the $B_{AG}$ and $B_{BG}$ by a carbon fraction factor equal to 0.5 Mg carbon/Mg d.m. The value of soil organic carbon (SOC) was obtained from the Italian greenhouse inventory report of 2023 [63].

##### Carbon Pools Related to Wetland

The carbon content that could be stored by a wetland depended on its type and could be sourced from Zhang et al. [66]. Table 5 contains a detailed description of carbon content in different wetland types.

##### Carbon Pools Related to Settlements, Water Body, and Other Land

The $C_{AG}$, $C_{BG}$, $C_{SOIL}$, and $C_{DOM}$ in the settlements were sourced from the study conducted by Babbar et al. [67]. The study considered the $C_{AG}$ as 2.00 Mg/ha, the $C_{BG}$ as 1.00 Mg/ha, the $C_{SOIL}$ as 5.00 Mg/ha, and the $C_{DOM}$ as 0. The carbon content in water bodies was sourced from the study conducted by Ma et al. [61], which stated the $C_{AG}$ as 2.00 Mg/ha, the $C_{BG}$ as 1.00 Mg/ha, the $C_{SOIL}$ as 10.00 Mg/ha, and the $C_{DOM}$ as 0. On the other hand, the other land class contained the carbon content found in bare soils, sourced from the study conducted by Zheng and Zheng [45], which considered 10.82 Mg/ha of the $C_{AG}$ and $C_{SOIL}$ as 15.88 Mg/ha. The $C_{BG}$ and $C_{DOM}$ were regarded as 0. Lastly, the economic value of CS/loss over time for a given parcel x was calculated by the InVEST model [68] as

(8) $$value\text{\_}seq_{x}=V\frac{s_{x}}{q-p}\sum_{t=0}^{q-p-1}\frac{1}{{\left(1+\frac{r}{100}\right)}^{t}{\left(1+\frac{c}{100}\right)}^{t}}$$

where *V* is the monetary value of carbon per Mg, $s_{x}$ is the amount of carbon in Mg sequestered or lost on parcel x, *q* is the future year, *p* is the present year, *r* is the annual market discount rate for the carbon price, and *c* is the annual rate of change in the carbon price. Table 6 represents the carbon pool table developed for the region of Calabria.

The valuation model for carbon pools at different times leveraged LULC information, the monetary value of carbon, and an annual discount rate. Utilizing LULC data spanning from 2000 to 2024, the model encompassed various categories according to the LULC classes chosen. The monetary value of elemental carbon was calculated to be 200 EUR/Mg, based on a CO_2_ price of EUR 60 per ton. This CO_2_ price is based on the considerations of the Organisation for Economic Co-operation and Development, a view widely supported by the International Monetary Fund [69]. The annual discount rate of 3% was applied to account for the time value of money. Notably, the model assumed an annual price change of 0%, reflecting a stable carbon price over time. The methodology involved calculating the carbon content within each LULC class, assigning a monetary value based on the fixed carbon price, and then discounting these values to their present value using the annual discount rate. The resulting present values were summed to determine the total valuation of carbon stocks for each year. This comprehensive approach considered the dynamic interplay between LULC changes and the economic value of carbon, providing insights into the financial implications of different carbon pools over time.

## 3. Results

### 3.1. LULC Scenario

**Figure 3:**
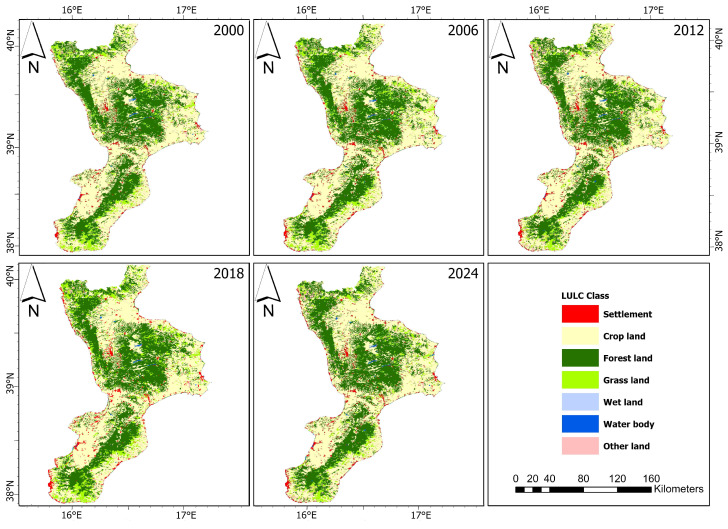
LULC maps of the AOI.

The spatial distribution of LULC is presented in Figure 3. Table 7 presents the LULC information for different years (2000, 2006, 2012, 2018, and 2024) in various categories. Notable trends can be observed over the years. Settlement areas have shown a steady increase from 446.66 km^2^ in 2000 to 560.05 km^2^ in 2018, with a further increase projected to 580.95 km^2^ by 2024. Cropland, on the other hand, experienced a fluctuating pattern, peaking at 7319.45 km^2^ in 2000 and gradually decreasing to 7121.24 km^2^ by 2024. Forest land also exhibited fluctuations, decreasing from 5536.18 km^2^ in 2000 to 5469.46 km^2^ in 2012, showing a slight increase by 2018, and then stabilizing around 5469.74 km^2^ by 2024. Grasslands saw an initial increase from 1482.16 km^2^ in 2000 to 1615.02 km^2^ in 2006, followed by a sharp decline to 1306.74 km^2^ in 2024. The wetland and water body areas remained relatively constant, with minimal changes, although water bodies increased slightly over the years. Other land categories showed significant fluctuations, with a sharp increase from 218.89 km^2^ in 2006 to 506.37 km^2^ in 2012 and predicted to increase further to 553.56 km^2^ by 2024. In summary, the data reflect dynamic changes in LULC, highlighting settlement expansion, cropland reduction, and notable variations in other land categories.

### 3.2. Validation of CA–Markov Model

The CA–Markov model used for LULC prediction was validated by comparing the predicted and reference LULC maps. The validation parameters are listed in Table 8; the validation produced different kappa parameters: $K_{std}$ kappa for standard, $K_{no}$ kappa for no information, $K_{loc}$ kappa for location, and $K_{locstrata}$ kappa for stratum-level location. $K_{std}$ computes the ratio of inaccurately allocated areas by chance to the correct assignments. $K_{no}$ represents the overall agreement between the predicted and reference map. $K_{loc}$ computes the spatial accuracy in the overall landscape by utilizing correct assignment values in each class between the predicted and reference maps. The quantification of the spatial accuracy within pre-identified strata is given by $K_{locstrata}$, and it measures how well the grid cells are located within the strata. The kappa parameters for the predicted LULC maps of 2012 and 2018 are 0.9656 and 0.9814, respectively, while the kappa parameters for location and stratum level location for 2012 are 0.9755 and 0.9755, and for 2018 are 0.9854 and 0.9854. Furthermore, the agreement/disagreement component validation analysis, reported in Table 9, evaluates the deviations between predicted and actual maps.

### 3.3. Carbon Scenario

**Figure 4:**
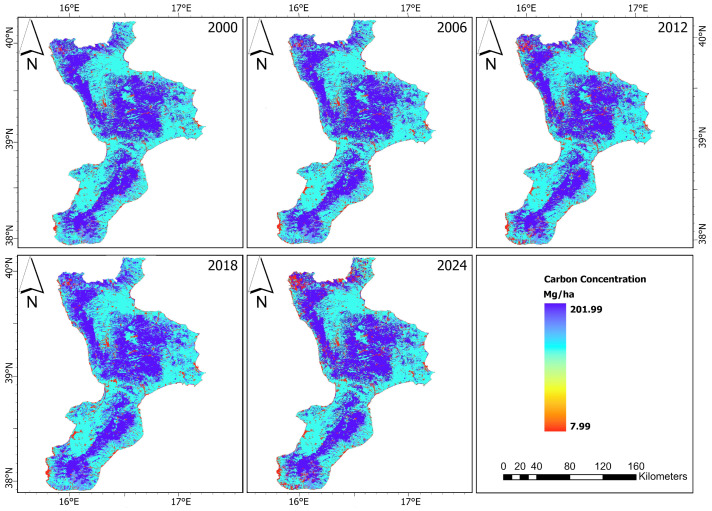
Carbon storage maps of the AOI.

The carbon concentration maps of the AOI are shown in Figure 4. Table 10 provides detailed information about the stored carbon, the sequestrated or lost carbon, and their associated economic values over the time periods from 2000 to 2024. The research findings reveal significant trends in carbon concentrations across various LULC classes during these intervals. In the initial assessment from 2000 to 2006, there was a marginal decrease in carbon concentration, amounting to 963,558.96 Mg, resulting in an economic loss of approximately EUR 192.57 million, based on a carbon price of 200 EUR/Mg, an annual market discount rate of 3%, and no annual price change. The decline in carbon concentration continued from 2006 to 2012, with a more substantial loss of 1,789,200.82 Mg, translating to an economic value loss of approximately EUR 357.57 million. Interestingly, the period from 2012 to 2018 marked a positive shift, with a modest increase in carbon concentration of 206,432.00 Mg. This increase was associated with an economic gain of approximately EUR 41.26 million. However, this trend did not persist, as the period from 2018 to 2024 saw a return to a decrease in carbon concentration, with a loss of 904,289.49 Mg, leading to an economic loss of about EUR 180.72 million.

## 4. Discussion

Effective soil management is pivotal for addressing global challenges, including supporting a growing and more affluent population, enhancing carbon sinks to mitigate excess atmospheric CO_2_, producing energy from biomass to improve energy security, and minimizing human encroachment into native ecosystems to lower anthropogenic CO_2_ emissions. Reducing anthropogenic greenhouse gas emissions to near zero is essential for climate stabilization [60]. Plants, through photosynthesis, capture and sequester CO_2_ in various plant tissues, both living and dead. While some of this captured carbon is eventually released back into the atmosphere via decomposition or combustion, a significant portion remains stored in soils or plant tissues over extended periods. This process, known as terrestrial CS, can be enhanced by altering land management practices to increase carbon storage in soils and plants, thereby offsetting atmospheric CO_2_ accumulation.

### 4.1. Methodological Performance and Limitations

The methodology employed in this study, integrating the InVEST model with RS and GEE, proved to be effective in detecting carbon content across diverse landscapes using satellite-sourced datasets and corresponding carbon pools. The InVEST model’s reliability in estimating regional carbon storage is well recognized, and its application in this study allowed for a comprehensive evaluation of carbon dynamics without disrupting natural habitats, which is particularly advantageous for protected and geographically inaccessible areas.

However, there are limitations to this approach. The accuracy of the results heavily depends on the quality and resolution of the input data, including the land use and land cover LULC datasets and the carbon pool values assigned to each LULC class. The LULC reclassification process, although streamlined for efficiency, may introduce some level of generalization that could affect the precision of the carbon stock estimations. For example, the classification of wetlands and the inclusion of additional land classes may not fully capture the variability in carbon storage potential across different types of landscapes. Another limitation is the reliance on the CA–Markov model for LULC predictions. While the CA–Markov model is robust and widely accepted, its predictive accuracy is constrained by the assumptions inherent in Markov chain processes and the quality of historical LULC data. The transition probability matrix, a core component of the model, was derived from average probabilities across several periods, which may not fully account for recent or future changes in land management practices, climate impacts, or policy shifts. The level of uncertainty in this study arises from several factors, including the resolution of satellite imagery, the accuracy of carbon pool estimates, and the assumptions underlying the predictive models. Although validation tests showed a substantial agreement between predicted and actual LULC data, as indicated by kappa statistics, some degree of uncertainty remains, particularly in the estimation of future LULC changes and their impact on carbon stocks.

### 4.2. Changes in LULC

This study’s innovative approach combines remotely sensed datasets, the InVEST model, and GEE to explore the CS potential of Calabrian landscapes over time. The spatial distribution of LULC over the years reveals notable trends, such as the increase in settlement areas from 446.66 km^2^ in 2000 to 560.05 km^2^ in 2018, projected to rise further to 580.95 km^2^ by 2024. Cropland has fluctuated, peaking at 7319.45 km^2^ in 2000 and gradually decreasing to 7121.24 km^2^ by 2024. Forest land has experienced fluctuations, decreasing from 5536.18 km^2^ in 2000 to 5469.46 km^2^ in 2012, with a slight recovery to 5488.18 km^2^ by 2018, followed by a small decline to 5469.74 km^2^ by 2024. Grasslands initially increased from 1482.16 km^2^ in 2000 to 1615.02 km^2^ in 2006 but then decreased significantly to 1306.74 km^2^ by 2024. Wetland areas showed a minor reduction from 1.25 km^2^ in 2000 to 0.47 km^2^ in 2006, remaining constant through 2024. Water bodies exhibited a gradual increase from 70.38 km^2^ in 2000 to 86.88 km^2^ by 2024. The “Other land” category saw a decline from 262.77 km^2^ in 2000 to 218.89 km^2^ in 2006, followed by a significant increase to 506.37 km^2^ by 2012 and a further rise to 553.56 km^2^ by 2024.

Table 7 presents the gains and losses in Land Use and Land Cover (LULC) classes between 2000 and 2024 across different time intervals: 2000–2006, 2006–2012, 2012–2018, and 2018–2024. Settlement areas showed a consistent increase from 446.66 km^2^ in 2000 to 547.42 km^2^ in 2006, with a further increase to 560.15 km^2^ in 2012 and 580.95 km^2^ by 2024. Cropland experienced a steady decline from 7319.45 km^2^ in 2000 to 7121.24 km^2^ in 2024, with the most significant decrease of 138.47 km^2^ occurring between 2000 and 2006. Forest land saw a decrease from 5536.18 km^2^ in 2000 to 5469.46 km^2^ in 2012, with a slight recovery between 2012 and 2018, increasing by 18.72 km^2^, but then stabilizing with a minor decline to 5469.74 km^2^ by 2024. Grassland displayed fluctuations, initially increasing by 132.86 km^2^ between 2000 and 2006, but then decreasing significantly by 261.51 km^2^ between 2006 and 2012, continuing to decrease by 22.55 km^2^ from 2018 to 2024. Wetland areas showed a minor reduction from 1.25 km^2^ in 2000 to 0.47 km^2^ in 2006, remaining constant through 2024. Water bodies exhibited a gradual increase from 70.38 km^2^ in 2000 to 86.88 km^2^ in 2024. The category of “Other land” saw a decline from 262.77 km^2^ in 2000 to 218.89 km^2^ in 2006, followed by a significant increase to 506.37 km^2^ by 2012, and further increased to 553.56 km^2^ by 2024.

The LULC changes in Calabria are driven by a complex interplay of agricultural practices, urbanization, climate change, policy interventions, socio-economic shifts, and natural disasters. Agricultural intensification, particularly in high-value crops, has led to the conversion of natural habitats, while agricultural abandonment in less accessible areas has resulted in natural reforestation. Urban expansion around cities like Reggio Calabria and Catanzaro, along with infrastructure projects, has further driven LULC changes by converting agricultural lands into residential and commercial areas. The region’s tourism industry, especially along the coast, has led to significant development, resulting in the loss of coastal wetlands and forests. Climate change has also played a crucial role by altering precipitation patterns and increasing the frequency of extreme weather events, which have accelerated changes in vegetation and land cover. European Union agricultural policies and forest conservation initiatives have influenced land use decisions, promoting certain types of agriculture while encouraging reforestation in other areas. Additionally, rural depopulation due to migration and economic shifts has led to the abandonment of agricultural lands, contributing to natural reforestation. Natural disasters like landslides and flooding, common in Calabria’s mountainous and coastal areas, have also significantly impacted land cover.

These changes in LULC indicate varying trends across different land use types, reflecting the impact of human activities and natural processes on the landscape. Understanding these trends is crucial for developing strategies to manage land resources effectively and to mitigate the effects of environmental changes. These changes directly impact CS capacities, as urban expansion typically leads to a decrease in natural vegetation cover, which is crucial for carbon storage. The stabilization of forest areas and the decline in grasslands suggest a shift in land use practices that may be influenced by economic and environmental factors. Similar trends have been observed in other European regions, where urbanization and agricultural intensification have led to changes in LULC and associated carbon dynamics. Terrestrial CS offers a safe and natural method for storing carbon, with additional benefits such as increased crop yields, improved soil water retention, and enhanced wildlife habitat. Implementing best management practices for terrestrial CS could conservatively sequester more than 0.5 petagrams (Pg) of carbon per year by 2040, potentially mitigating 6–23% of emissions and storing up to 40 Pg of carbon by 2100 [70]. Options for terrestrial CS include the restoration of degraded lands, afforestation, reforestation, rangeland improvement, better tillage practices, and wetland restoration. Planting trees in cleared areas and abandoned farmlands can significantly increase carbon storage per ha compared to grasslands.

### 4.3. Changes in Carbon Stocks

The research findings reveal significant trends in carbon concentrations across various LULC classes over the specified periods. From 2000 to 2006, there was a marginal decrease in carbon concentration by 963,558.96 Mg, translating to an economic loss of approximately EUR 192.57 million. This decrease is equivalent to 3,532,052.52 Mg of CO_2_ emissions. The decline continued from 2006 to 2012, with a loss of 1,789,200.82 Mg of carbon, valued at about EUR 357.57 million, which corresponds to 6,558,069.68 Mg of CO_2_. However, between 2012 and 2018, a modest increase in carbon concentration (206,432.00 Mg) was observed, resulting in an economic gain of EUR 41.26 million. This increase is equivalent to 756,919.47 Mg of CO_2_. Unfortunately, from 2018 to 2024, the trend reversed again, with a decrease in carbon concentration of 904,289.49 Mg, equating to an economic loss of EUR 180.72 million, which is equivalent to 3,315,063.79 Mg of CO_2_ emissions. These findings are consistent with studies conducted in other regions [7,45], which also report fluctuations in carbon stocks due to varying land use practices and environmental conditions. The observed changes in carbon stocks in Calabria reflect broader global patterns of CS and loss, underscoring the importance of effective land management practices to mitigate carbon emissions and enhance carbon storage.

### 4.4. Relationship between Carbon and the Economy

The economic implications of carbon stock changes are evident in the study’s findings, with significant financial losses observed during periods of high deforestation and land degradation. In the context of biological carbon, there exists monetary value in the sequestering role of LULCs through carbon credits and markets. By quantifying the carbon storage capacity of different land uses, landowners can participate in carbon markets. The payment of environmental schemes remunerates landowners for maintaining or increasing CS in their lands; hence they create economic benefits for forest landholders, grassland landholders, and other landholders in carbon-rich ecosystems [71]. The economic costs to mitigate climate change are substantially linked to LULC. Reforestation and afforestation and improved agricultural practices leading to CS are cost-efficient ways to reduce net greenhouse gas emissions. These strategies can also bring co-benefits, such as biodiversity conservation, water regulation, and soil fertility improvement, which have additional economic value [72]. The economic value of CS highlights the importance of strategic land management practices that promote carbon storage and reduce emissions. In regions like Calabria, where agriculture and urban development compete with forest conservation, policies that incentivize sustainable land use practices are crucial.

### 4.5. Interaction between Carbon and Environmental Factors

This research also reveals the spatial distribution of carbon stocks and highlights the importance of specific land management practices in enhancing CS. For instance, afforestation and reforestation efforts in degraded lands not only restore ecological balance but also contribute significantly to CS. The conversion of abandoned farmlands to forests can increase carbon storage, demonstrating the potential of strategic land use changes in climate change mitigation. Although there is debate about the exact amounts of carbon stored and emitted from terrestrial ecosystems, there is consensus that substantial quantities of carbon are held in world soils, with emissions from LULCC being the second-largest anthropogenic source of carbon after fossil fuel combustion [60]. Considering the significant amounts of organic carbon stored in soils, it is crucial to manage this resource to either mitigate or exacerbate climate change. The global pool of SOC in the top 30 cm of soils is estimated to be between 684 and 724 Pg of carbon. Small changes in this large stock could significantly impact future atmospheric CO_2_ concentrations. If SOC stocks decline due to poor management practices or climate change, additional CO_2_ will be released into the atmosphere, exacerbating climate change. Conversely, management practices that increase SOC over large areas can slow the rise in atmospheric CO_2_ and help mitigate climate change. Key questions remain about the response of soils to climate change, including the temperature sensitivity of SOC, the balance between increased carbon input from plant production and increased losses due to decomposition, and the interactions between climate change and other global changes such as water balance and atmospheric composition. The uncertainty in soil carbon estimates remains high [73], likely due to uncertainties in input data, model types, and resolution. Climate change is expected to influence both the quantity and quality of carbon entering soils from plant inputs and its decomposition rate. For instance, higher atmospheric CO_2_ levels could boost plant productivity and carbon inputs from litter and roots, although plant growth will also be affected by temperature and water availability. These influences can be positive or negative, depending on whether climate change alleviates current growth constraints or imposes new ones. Environmental factors, such as temperature, precipitation, and soil type, play a critical role in determining CS potential. At both the global and sub-regional levels, the amount and vertical distribution of organic carbon in soil, as well as its storage, are greatly influenced by climate conditions, especially the annual average temperature and precipitation, which influence the rate at which organic carbon breaks down, as well as the amount of carbon that enters the soil [74]. These climatic impacts result in variations in the content of SOC across different locations. For example, low temperatures and wet conditions prevent organic matter from decomposing and cause organic carbon to build up; as a result, compared to hot and dry climates, SOC contents are often higher in cold and damp locations [75]. In many terrestrial habitats, precipitation controls the primary productivity of plants and, consequently, the amount of carbon incorporated into the soil [76]. Furthermore, moisture promotes the development of mineral surfaces that stabilize soil organic carbon by accelerating the weathering of the parent rock [77]. It also frequently results in soil acidity, which slows the breakdown of organic matter [78]. Since the complex molecular properties of organic matter are extremely sensitive to temperature changes, variations in temperature and moisture levels impact microbial and biotic activity, which in turn causes changes in the microbial degradation of organic matter [79]. Many studies have found that, despite the various restrictions on this relationship, higher air temperatures tend to accelerate the decomposition of organic matter and increase organic carbon losses, whereas lower temperatures limit this process and subsequently raise organic carbon concentrations [74,80]. Understanding these interactions is essential for developing effective land management strategies that enhance carbon storage while adapting to changing environmental conditions.

The findings of this study are consistent with previous research on the impact of LULC changes on carbon storage and sequestration. Similar to the study conducted by Wang et al. [33] in the West Liaohe River Basin, which demonstrated that optimizing LULC through the expansion of ecological lands such as forests and grasslands can significantly enhance carbon storage and reduce emissions, our results also highlight the critical role of strategic LULC management in achieving carbon neutrality. Furthermore, our study aligns with the research conducted by Babbar et al. [67] on the Sariska Tiger Reserve, which identified substantial carbon losses due to deforestation but also emphasized the potential for carbon recovery through targeted afforestation and reforestation efforts. Furthermore, the study Wang et al. [32] emphasizes the critical role of LULC in influencing carbon stocks and Ecosystem Services. Additionally, it highlights the importance of balancing land development with ecological conservation to manage carbon storage effectively. These parallels across different studies reinforce the robustness of the findings of this study and their relevance to broader efforts in managing carbon stocks and mitigating climate change.

## 5. Conclusions

The methodology employed in this study has leveraged the InVEST model, which is a robust approach for detecting carbon content using information about landscapes obtained from satellite imagery and corresponding carbon pools. Our study highlights significant changes in LULC over the past two decades, directly impacting CS capacities and leading to economic implications. Settlement, bare land expansion, and the reduction in forest and grassland areas have notably decreased carbon storage, while agricultural shifts have further influenced these dynamics. Though minor recoveries in carbon storage were observed between 2012 and 2018, overall losses underscore the importance of strategic land management. We recommend the implementation of sustainable land use practices, such as forest conservation, restoration, and agroforestry, to mitigate carbon losses and enhance CS. Future research should focus on refining land classification methods and developing strategies to balance economic growth with environmental sustainability. These efforts will be critical in supporting climate mitigation and promoting long-term ecological health. Notably, the methodology is tailored for the Calabria region, incorporating a custom carbon pool table that accounts for the prevalent tree species and crop types in the area’ therefore adapting this methodology to other study areas requires modifying the carbon pools table, growing stock, and corresponding scaling parameters according to the specific characteristics of the new study area. While this approach enhances the accuracy of estimations and is both time-efficient and cost-effective, there is room for improvement, particularly in refining the classification of wetlands and the inclusion of more tree species to precisely account for variations in carbon storage. We believe that the outcomes of this study could reinforce the importance of rigorous, transparent, and economically integrated carbon accounting methods. For Verified Carbon Standards [81] and other frameworks, these findings suggest the need for continuous improvement in monitoring, economic valuation, and methodological transparency to enhance the credibility and effectiveness of carbon markets. By incorporating these insights, carbon accounting methods can better support climate mitigation efforts and attract sustainable investments.

## Figures and Tables

**Table 1:** Description of land use and land cover classes obtained by merging Corine land cover classes using a reclassify tool in Google Earth Engine.

LULC Class	Description	Corine Landcover Classes Combined
Settlement	Areas dominated by infrastructure	(1) Continuous urban fabric; (2) Discontinuous urban fabric; (3) Industrial or commercial units; (4) Road and rail networks and land; (5) Port areas; (6) Airports; (7) Mineral extractions sites; (8) Dump sites; (9) Construction sites; (10) Green urban areas; (11) Sport and leisure facilities.
Crop land	Areas where farming is performed	(1) Non-irrigated arable land; (2) Permanently irrigated land; (3) Rice fields; (4) Vineyards; (5) Fruit trees and berry plantations; (6) Olive grooves; (7) Annual crops associated with permanent crops; (8) Complex cultivation patterns; (9) Land principally occupied by agriculture, with significant area of natural vegetation.
Forest land	Areas with high-density of trees	(1) Agro-forestry areas; (2) Broad-leaved forest; (3) Coniferous forest; (4) Mixed forest.
Grassland	Green areas with no or sparse tree cover	(1) Natural grasslands; (2) Moors and heathland; (3) Sclerophyllous vegetation; (4) Transitional woodland-shrub; (5) Pastures; (6) Sparse vegetative areas.
Wetland	Damped or wet areas	(1) Inland marshes; (2) Peat bogs; (3) Salt marshes; (4) Salines; (5) Intertidal flats.
Water body	Areas partially or totally covered by water	(1) Water courses; (2) water bodies; (3) Coastal lagoons; (4) Estuaries; (5) Sea and ocean.
Other land	Areas with no vegetation	(1) Beaches, dunes, sands; (2) Bare soil and rocks; (3) Burnt areas; (4) Glaciers and perpetual snow.

**Table 2:** Average transition probability matrix.

	Settlement	Crop Land	Forest Land	Grassland	Wet Land	Water Body	Other Land
Settlement	0.9862	0.0081	0.0019	0.0028	0.0000	0.0005	0.0004
Crop land	0.0058	0.9860	0.0019	0.0058	0.0000	0.0001	0.0003
Forest land	0.0001	0.0035	0.9872	0.0083	0.0000	0.0001	0.0007
Grassland	0.0004	0.0134	0.0253	0.8977	0.0000	0.0024	0.0607
Wet land	0.0000	0.0000	0.0000	0.1070	0.7920	0.1009	0.0000
Water body	0.0009	0.0008	0.0000	0.0000	0.0000	0.9963	0.0020
Other land	0.0007	0.0086	0.0083	0.0688	0.0000	0.0006	0.9131

**Table 3:** Factors used in scaling growing stock ($G_{S}$).

	Biomass Expansion Factor	Wood Basic Density	Carbon Fraction	Root to Shoot Ratio	Average Mortality Rate
Forest Tree Type	$\left(B_{\mathrm{EF}}\right)$	$\left(W_{\mathrm{BD}}\right)$	$\left(C_{F}\right)$	(R)	$\left(A_{\mathrm{MR}}\right)$
European beech	1.36	0.61	0.47	0.20	0.0177
Chestnut	1.33	0.49	0.47	0.28	0.0177
Turkey oak	1.45	0.69	0.47	0.24	0.0177
Larches	1.22	0.56	0.47	0.29	0.0177
Mean value	1.34	0.58	0.47	0.25	0.0177

**Table 4:** Carbon content (Megagram/hectare) in major woody crops grown in Calabria.

Crop Type	Maturity Cycle (Years)	Carbon above Ground	Carbon below Ground
		$\left(C_{\mathrm{AG}}\right)$	$\left(C_{\mathrm{BG}}\right)$
Olive	50	9.13	2.60
Vineyards (wine grapes)	20	5.6	4.46
Vineyards (other)	30	5.62	4.48
Orchards	25	8.91	5.75
Other fruits	20	8.90	5.73
Mean value	-	7.63	4.60

**Table 5:** Carbon content in different wetland types (megagrams/hectare).

Wetland Type	Carbon above Ground	Carbon below Ground	Soil Carbon	Dead Organic Matter Carbon
	($C_{\mathrm{AG}}$)	($C_{\mathrm{BG}}$)	($C_{\mathrm{SOIL}}$)	($C_{\mathrm{DOM}}$)
Swamp	18.00	18.50	161.50	10.00
Lake related wetland	6.00	19.50	11.50	0
River related wetland	6.00	32.00	71.50	0
Beach related wetland	39.00	19.50	62.00	10.00
Mangrove wetland	48.50	106.00	206.00	3.00
Pond related wetland	18.00	6.50	17.00	0
Mean value	22.58	33.66	88.25	3.83

**Table 6:** Carbon pool table (megagram/hectare) served as input to the InVEST model.

	Carbon above Ground	Carbon below Ground	Soil Carbon	Dead Organic Matter Carbon
LULC Class	($C_{\mathrm{AG}}$)	($C_{\mathrm{BG}}$)	($C_{\mathrm{SOIL}}$)	($C_{\mathrm{DOM}}$)
Settlement	2	1	5	0
Crop land	8	5	46	1
Forest land	93	17	83	9
Grassland	1	5	66	0
Wet land	23	34	88	4
Water body	2	1	10	0
Other land	2	0	16	0

**Table 7:** Gains and losses in LULC classes between 2000 and 2006, 2006 and 2012, 2012 and 2018, and 2018 and 2024.

LULC Classes	LULC 2000	LULC 2006	LULC 2012	LULC 2018	LULC 2024
	[km^2^] (%)	[km^2^] (%)	[km^2^] (%)	[km^2^] (%)	[km^2^] (%)
Settlement	446.66 (2.95%)	547.42 (3.62%)	560.15 (3.70%)	560.05 (3.70%)	580.95 (3.84%)
Crop land	7319.45 (48.41%)	7180.98 (47.50%)	7151.97 (47.30%)	7151.02 (47.30%)	7121.24 (47.10%)
Forest land	5536.18 (36.62%)	5482.54 (36.26%)	5469.46 (36.18%)	5488.18 (36.30%)	5469.74 (36.18%)
Grassland	1482.16 (9.80%)	1615.02 (10.68%)	1353.51 (8.95%)	1329.28 (8.79%)	1306.74 (8.64%)
Wet land	1.25 (0.01%)	0.47 (0.00%)	0.47 (0.00%)	0.47 (0.00%)	0.47 (0.00%)
Water body	70.38 (0.47%)	73.52 (0.49%)	76.92 (0.51%)	83.84 (0.55%)	86.88 (0.57%)
Other land	262.77 (1.74%)	218.89 (1.45%)	506.37 (3.35%)	505.99 (3.35%)	553.56 (3.66%)

**Table 8:** Validation of LULC maps with kappa parameters using the validation module in TerrSet. The first two rows refer to LULC maps for the years 2012 and 2018, respectively.

Type	Year	$K_{\mathrm{Std}}$	$K_{\mathrm{no}}$	$K_{\mathrm{loc}}$	$K_{\mathrm{locstrata}}$
LULC	2012	0.9656	0.9773	0.9755	0.9755
LULC	2018	0.9814	0.9877	0.9854	0.9854

**Table 9:** Results of validation analysis (agreement/disagreement component values) in TerrSet 2020 for the years 2012 and 2018.

Agreement/Disagreement	LULC 2012	LULC 2018
Agreement due to chance	0.1250	0.1250
Agreement due to quantity	0.2976	0.2973
Agreement at stratum level	0.0000	0.0000
Agreement at gridcell level	0.5575	0.5669
Disagreement at gridcell level	0.0140	0.0084
Disagreement at stratum level	0.0000	0.0000
Disagreement due to quantity	0.0059	0.0023

**Table 10:** Stored carbon, sequestration and losses between 2000 and 2006, 2006 and 2012, 2012 and 2018, and 2018 and 2024.

Year	Stored Carbon	Economic Value of Stored Carbon	Variation	Equivalent Units of CO_2_	Economic Value of Variation
	[Mg]	[Million EUR]	[MgC]	[Mg CO_2_]	[Million EUR]
2000	167,359,466.90	33,471.89			
2006	166,395,908.00	33,279.18	−963,558.96	−3,532,052.52	−192.57
2012	164,606,707.10	32,921.34	−1,789,200.82	−6,558,069.68	−357.57
2018	164,813,139.20	32,962.63	+206,432.00	+756,919.47	+41.26
2024	164,202,650.36	32,840.53	−904,289.49	−3,315,063.79	−180.72

## Data Availability

All relevant data are included in the manuscript.

## Abbreviations

The following abbreviations are used in this manuscript:

AOI	Area of Interest
$A_{MR}$	Average Mortality Rate
$B_{AG}$	Above Ground Biomass
$B_{BG}$	Blow Ground Biomass
$B_{EF}$	Biomass Expansion Factor
CA	Cellular Automata
CA–Markov	Cellular Automata-Markov Chain
$C_{AG}$	Carbon Above Ground
$C_{BG}$	Carbon Blow Ground
$C_{DOM}$	Deadwood Organic Matter Carbon
$C_{F}$	Carbon Fraction
CLC	Corine Land Cover
CO_2_	Carbon dioxide
CS	Carbon Sequestration
ESs	Ecosystem Services
FREL	Forest Reference Emission Level
GEE	Google Earth Engine
GIS	Geographic Information System
$G_{S}$	Growing Stock
ha	Hectare
InVEST	Integrated Valuation of Ecosystem Services and Tradeoffs
IPCC	Intergovernmental Panel on Climate Change
LULC	Land Use Land Cover
LULUCF	Land Use Land Use Change and Forestry
Mg	Megagram
MgC	Megagram Carbon
MRV	Monitoring, Reporting, and Verifying
Pg	Petagram
REDD	Reducing Emissions from Deforestation and Forest Degradation
RS	Remote Sensing
SOC	Soil Organic Carbon
Tg	Teragram
UNFCCC	United Nations Framework Convention on Climate Change
$W_{BD}$	Wood Basic Density
